# Moral Emotions and Social Economic Games in Paranoia

**DOI:** 10.3389/fpsyt.2018.00615

**Published:** 2018-11-21

**Authors:** George Savulich, Hannah Jeanes, Nicole Rossides, Sahaj Kaur, Alice Zacharia, Trevor W. Robbins, Barbara J. Sahakian

**Affiliations:** ^1^Department of Psychiatry, University of Cambridge, School of Clinical Medicine, Cambridge, United Kingdom; ^2^Behavioural and Clinical Neuroscience Institute, University of Cambridge, Cambridge, United Kingdom; ^3^Department of Psychology, University of Cambridge, Cambridge, United Kingdom

**Keywords:** paranoia, social cognition, moral emotions, economic games, delusions

## Abstract

Impaired social cognitive processes are putative psychological mechanisms implicated in the formation and maintenance of paranoid beliefs. Paranoia denotes unfounded fears about the hostile intentions of others and is prevalent in a significant proportion of the general population. We investigated social cognition in healthy participants selectively recruited to have a broad occurrence of paranoid thinking (*n* = 89). Participants completed a novel computerized task of moral emotions and two social economic exchange games (Prisoner's Dilemma, Ultimatum Game) from the EMOTICOM neuropsychological test battery. Regression analyses revealed that delusional ideation predicted shameful feelings when the victim of deliberate harm by another person. Cooperative behavior on the Prisoner's Dilemma was greatest when the participant and opponent contributed equally to joint earnings. Participants demonstrated significantly more punishment behavior when contributions were unequal and stole more from the opponent using a suspicious strategy of gameplay. In addition, paranoid thinking was positively associated with more stealing from the cooperative opponent. On the Ultimatum Game, participants accepted significantly more unequal offers when the opponent contributed more and sensitivity to fairness was greatest when the participant contributed more. These data demonstrate that delusional ideation predicts a maladaptive emotional response to interpersonal harm and that paranoid thinking may lead to reduced cooperation toward mutual reward. The effects of paranoia on moral emotions and pro-social behavior at more severe levels of persecutory thinking warrant further investigation.

## Introduction

Impaired social cognition is a key feature of schizophrenia, with deficits typically found in emotion identification, experience sharing and emotional responding (1). These impairments are one pathway to a first episode of psychosis (2) and strongly predict functional and social outcomes in psychotic disorders [(3, 4)]. In patients with schizophrenia, impaired social cognitive processes have shown to account for a larger proportion of the variance in community functioning than non-social cognitive impairments (5). Social cognitive impairments have also shown to lead to more difficulty inferring the mental states of others, including their beliefs and intentions (1, 6).

Paranoia is characterized by unfounded fears about the harmful intentions of others (7). Social cognition is highly relevant to paranoid thinking, as hostile perceptions during social interactions are likely to precipitate threat beliefs and related distress. Paranoid thinking is symptomatic of psychosis and also prevalent in 10–15% of the general population (8). Paranoia is associated with several cognitive and affective processes implicated in the formation and maintenance of a persecutory delusion (9, 10). “Jumping to conclusions” (JTC) is a reliably established probabilistic reasoning bias whereby deluded patients use less information to make a decision compared with healthy controls [e.g., (11)]. Individuals with non-clinical paranoia have also shown evidence of cognitive biases similar to those with persecutory delusions. For example, healthy individuals with elevated trait paranoia have shown to interpret emotionally ambiguous information in a paranoid manner (12), an effect that matched symptoms in patients with psychosis (13). Studies of non-clinical paranoid experiences are therefore important to inform our understanding of those presenting clinically.

Studies of social cognition in schizophrenia have primarily focused on impairments in emotion perception, theory of mind and attributional style (1, 14). Deficits in theory of mind and heightened social anxiety have shown to make independent contributions to the development of paranoia, thus raising the possibility of distinct cognitive and emotional pathways (15). Social anxiety is also related to negative symptoms and self-stigma in schizophrenia, often leading to expectation of embarrassment or rejection (15, 16). It has been proposed that persecutory delusions are triggered by interpersonal stress leading to complex interactions between reduced mentalizing abilities, a vulnerable self-concept and over-activation of the threat/protection system (17). Paranoia is then reinforced by cognitive biases supporting inflexible beliefs about being at risk of harm by others (e.g., JTC; interpretation bias; bias against disconfimatory information) [see (18) for a review]. Studies of attributional bias have further shown that patients with persecutory delusions make externalizing attributions for negative events by blaming others, which serves to protect the self (19–21). However, these studies typically use questionnaire measures that present hypothetical positive/negative situations without measuring emotional responses or differentiating the intention of the agent of action. We thus used a novel “Moral Emotions” task to investigate emotional responses when both the *victim* and *victimiser* of *accidental* (unintentional) and *deliberate* (intentional) harm in cartoon scenarios depicting interpersonal behavior (22). Guilt and shame are two moral emotions associated with a range of psychological disorders (23). Guilt develops in recognition of oneself as an agent of a negative outcome for another person, whereas shame reflects an emotional appraisal of oneself as personally inadequate, usually following judgement, criticism, or humiliation by others (24). Shame increases paranoia following a stressful life event (25) and is associated with anxiety-related processes in the general population (26). Moral emotions are therefore likely to be compromised in healthy individuals with high levels of paranoia and predicted by variation in different traits (e.g., paranoia, anxiety) depending on the intention of another person when harmed.

Social decision-making is another cognitive process influenced by the inferred knowledge and intentions of others (27, 28). Economic exchange games, such as the “Prisoner's Dilemma” (29) and “Ultimatum Game” (30), are established interactive paradigms for assessing cooperation, sensitivity to fairness and the tendency to inflict punishment. These games involve choosing to split sums of money based on the player's contribution, the opponent's behavior and the amount proposed. Paranoia has shown an association with distrust-based behavior (expecting a competitive opponent), but not greed-based behavior (exploiting a cooperative opponent), when playing the Prisoner's Dilemma (31). Paranoia has also shown an association with more attributions of harmful intent for both fair and unfair dictators on the “Dictator Game” (32). When playing the Ultimatum Game, healthy participants consistently forfeit their own gains when offers are deemed considerably unfair [e.g., below 30%; (33)], which is thought to reflect heightened sensitivity to fairness (34) or a desire to punish socially unacceptable behavior (35). Patients with schizophrenia are less strategic and have shown to accept more unfair offers and reject more fair offers compared with healthy controls (36). However, others have found higher rejection of unfair compared with fair offers in patients with schizophrenia (37, 38) as well as no significant differences in acceptance rates compared with controls (39). It is possible that behavioral performance is motivated by distrust about the opponent's intentions (i.e., predicting that the opponent will always defect, despite minimizing mutual outcomes), but that sensitivity to fairness remains relatively intact or relates to the severity of symptoms in schizophrenia (for example, is impaired in those with negative symptoms).

We investigated social cognition in the general population reporting a broad occurrence of paranoid thinking. Specifically, we examined the role of paranoia and other traits relevant to psychosis on moral emotional processing and social decision-making, two processes requiring the ability to infer the mental states of others. As paranoia denotes fears about the harmful intentions of others (7), we expected that high levels of paranoia would alter both emotional and cognitive processing during three tasks involving the perception of other's intentions toward the self. Tasks were selected from EMOTICOM (22), a novel neuropsychological test battery for assessing affective domains. On the basis of models indicating a weakened sense of self in those with paranoia [e.g., (17)], we firstly hypothesized that paranoid thinking would predict shameful feelings when the victim of intentional (but not unintentional) harm by another person on the Moral Emotions task. Secondly, we hypothesized that in line with previous research [e.g., (31)], distrust-based punishment behavior would be greatest when playing against a suspicious opponent on the Prisoner's Dilemma, and that choice to compete (stealing) would be associated with paranoid thinking. Finally, we hypothesized that acceptance rates would increase as offers became increasingly fair on the Ultimatum Game, consistent with preserved sensitivity to fairness [e.g., (37, 39)].

## Materials and methods

### Participants

Eighty-nine participants were recruited from internal mailing of a volunteer panel at the University of Cambridge and advertisements in the local Cambridgeshire area. Inclusion criteria were fluency in English; not currently taking any psychiatric medication or receiving psychological treatment; and not having a current or past psychiatric diagnosis. All participants were screened on these criteria using the Mini-International Neuropsychiatric Interview (40). Participants were selectively recruited to have a wide range of scores of the Green Paranoid Thoughts Scale [GPTS; (41)] to capture naturally occurring paranoid thinking in the general population. In order to reduce multicollinearity between predictor variables potentially entered in regression analyses (up to seven trait measures, see below), a sample size of at least 80 was determined to ensure that there were at least 10 times as many observations from the sample.

### Questionnaire measures

The National Adult Reading test [NART; (42)] is a 50-item estimate of premorbid intelligence. Participants are instructed to read aloud 50 words of atypical phonemic pronunciation. Higher scores (0–50) indicate more correct responses (i.e., higher intelligence).

The Green Paranoid Thoughts Scale [GPTS; (41)] is a 32-item multidimensional measure of paranoid thinking including thoughts of persecution and ideas of reference. Participants indicate thoughts that they might have had about others in the last month using a 5-point Likert scale (1 = not at all to 5 = totally). Higher scores (0–160) indicate more paranoid thinking.

The Paranoia Scale [PS; (43)] is a 20-item measure of trait paranoia. Participants indicate thoughts about themselves and others using a 5-point Likert scale (1 = not at all to 5 = totally). Higher scores (0–100) indicate more trait paranoia.

Peters' Delusions Inventory [PDI-21; (44)] is a 21-item multidimensional measure of delusional ideation (including beliefs and vivid mental experiences). Participants first circle “yes/no” questions about experiences they might have had. For “yes” answers, participants rate how distressing, preoccupying, and true they believe each experience to be using 5-point Likert scales (1 = not at all distressing to 5 = very distressing; 1 = hardly ever think about it to 5 = think about it all the time; 1 = don't believe it's true to 5 = believe it is absolutely true). Higher scores (0–336) indicate more delusional ideation.

The Cardiff Anomalous Perceptions Scale [CAPS; (45)] is a 32-item measure of anomalous perceptions. Participants answer “yes/no” questions about sensations and perceptions that they may have experienced. Higher scores (0–32) indicate more anomalous perceptions.

The Spielberger Trait Anxiety Scale [STAI-State; (46)] is a 20-item of trait anxiety. Participants rate statements in relation to how they usually feel using a 4-point Likert scale (1 = not at all to 4 = very much so). Higher scores (0–80) indicate more trait anxiety.

The Beck Depression Inventory [BDI-II; (47)] is a 21-item of depression. Participants read statements and circle answers corresponding with how they have been feeling in the past 2 weeks. Higher scores (0–63) indicate more depression.

The Cognitive Flexibility Scale [CF; (48)] is a 12-item measure of cognitive flexibility (i.e., awareness of situational alternatives). Participants rate statements about their beliefs, feelings, and behaviors using a 6-point Likert scale (1 = I strongly disagree to 6 = I strongly agree). Higher scores (0–72) indicate more cognitive flexibility.

### *EMOTICOM* measures

#### Moral emotions task

**Figure 1:**
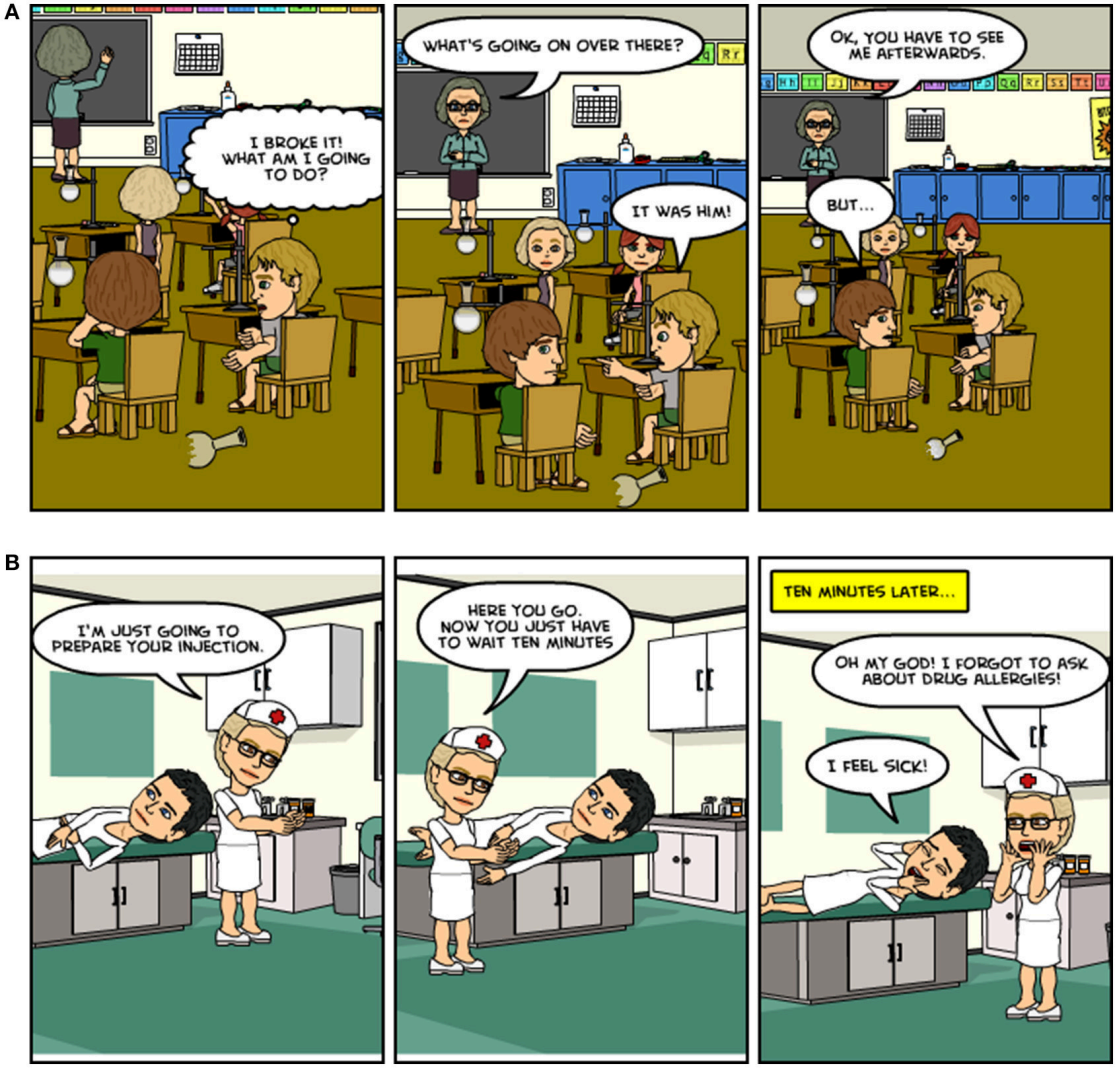
**(A)** An example of a scene from the EMOTICOM Moral Emotions task depicting both the victim and victimiser of deliberate harm**. (B)** An example of a scene from the EMOTICOM Moral Emotions task depicting both the victim and victimiser of accidental harm.

The Moral Emotions task measures moral responses to intentional and unintentional harmful actions by another person (22). Participants were presented with moral scenarios using cartoons. Half of the scenarios depict deliberate harm by another person (Figure 1A), whereas the other half depicts accidental harm by another person (Figure 1B) (both leading to a negative outcome). Participants were asked to rate how much shame and guilt that they have in each scenario as both the victimiser (the person who commits the action) and victim (the person who experiences the consequences). Higher average ratings indicate more shame and guilt.

#### Prisoner's dilemma

**Figure 2:**
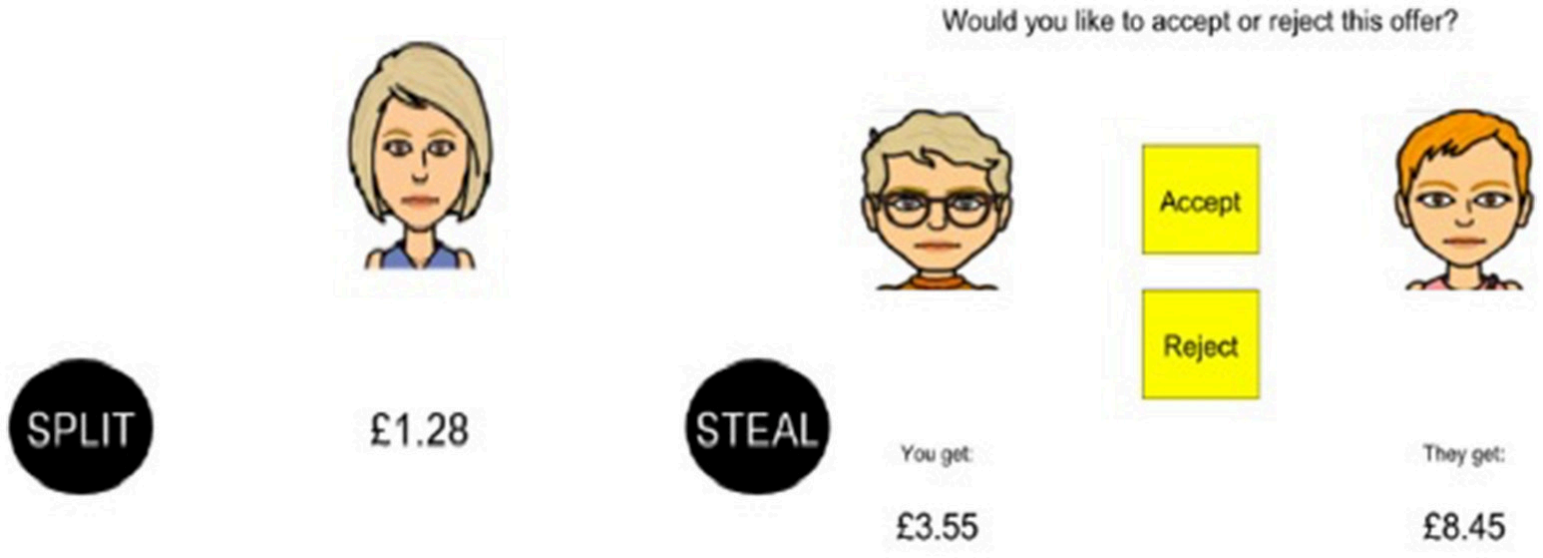
Examples of offers from the Prisoner's Dilemma (left) and Ultimatum Game (right).

The Prisoner's Dilemma assesses cooperation with an opponent (29). Participants were first asked to compete with an avatar by pressing the space bar, as quickly as possible, to fill a jar with coins. Each trial is manipulated so that the participant wins more coins, the opponent wins more coins or both the participant and the opponent win the same amount of coins. Earnings are then combined and the participant is instructed to either split or steal the total sum. Participants are told that if they (the participant and the opponent) both split, then they each get half the money, and if they both steal, then they each get nothing (Figure 2). However, if the participant steals and the opponent splits, then the participant gets the total earnings and the opponent gets nothing. Participants face three different opponent strategies throughout the game: suspicious (tit for tat, but starts with steal), tit for two tats (starts with split, then changes behavior after the player steals two times consecutively) and cooperative (always splits).

#### Ultimatum game

The Ultimatum Game (30) assesses fairness sensitivity and the tendency to inflict punishment following an unfair offer. Participants and an avatar first earn money by independently uncovering three out of nine yellow ovals; ovals that turn black reveal £3 and ovals that turn red earn nothing. Similar to Prisoner's Dilemma, each trial is manipulated so that the participant wins more money, the opponent wins more money or both the participant and the opponent win the same amount of money. Earnings are then combined and the participant is told whether or not they or the opponent will decide how the total sum is split. If the opponent decides, then the participant gets the choice either to accept or reject their offer (Figure 2). Offers have seven levels of fairness ranging from very fair (50:50%) to increasingly unfair (10:90%). If the participant accepts, then they each get the allotted amount, and if they reject, then they both get nothing.

### Procedure

This study received full ethical approval from the University of Cambridge Psychology Research Ethics committee (reference: Pre.2015.046). Participants meeting inclusion criteria (after telephone screening) were invited to attend a single session at the University of Cambridge Behavioral and Clinical Neuroscience Institute. Participants first provided written informed consent, followed by basic demographic information and an estimate of premorbid intelligence. Participants then completed EMOTICOM measures using a touch screen laptop (Dell XT3) in a counterbalanced order using a Latin-square design. The EMOTICOM task battery was delivered using PsychoPy. Participants then completed the questionnaire measures; questionnaires were always administered after the tasks to reduce paranoia-related demand characteristics. Participants were thanked and paid for their time.

## Results

### Participant characteristics

Participant characteristics for the whole sample (*n* = 89) are presented in Table 1. The mean GPTS score was similar to those previously reported in other samples from the general population recruited using this scale, *M* = 53.57, *SD* = 19.91, range = 32–111 (12, 41).

**Table 1:** Demographic and trait measures for the whole sample (means and standard deviations).

	***n* = 89**
**DEMOGRAPHIC MEASURES**	
Age (years)	22.29 (±5.22)
Gender (male: female)	35 M: 54 F
Intelligence (NART)	110.72 (±9.44)
**TRAIT MEASURES**	
Paranoid thinking (GPTS)	53.57 (±19.91)
Paranoia (PS)	39.18 (±11.01)
Delusional ideation (PDI)	39.92 (±28.19)
Anomalous perceptions (CAPS)	4.62 (±4.34)
Anxiety (STAI-Trait)	10.01 (±3.53)
Depression (BDI-II)	6.11 (±5.65)
Cognitive flexibility (CF)	56.96 (±6.28)

*NART, National Adult Reading Test; GPTS, Green Paranoid Thoughts Scale; PS, Paranoia Scale; PDI, Peters' Delusions Inventory; CAPS, Cardiff Perceptions Inventory; STAI-Trait, Spielberger Trait Anxiety Inventory; BDI-II, Beck Depression Inventory; CF, Cognitive Flexibility Scale*.

### Moral emotions task

#### Regression analyses

Personality trait measures were first correlated with moral emotions. Only variables with more than one significant association were entered as predictor variables in a hierarchical multiple regression analysis, with paranoia and psychosis measures entered into Block 1 and anxiety, depression, and cognitive flexibility measures entered into Block 2. This order of entry controls for highly correlated and conceptually similar variables (e.g., anxiety; Block 2) without corrupting the predictors of *a priori* interest (e.g., paranoid thinking; Block 1) (49).

#### Victimiser of harm (deliberate and accidental)

Moral emotions were not significantly associated with any trait measure when the victimiser of deliberate or accidental harm (all *p*'s > 0.07). Average ratings for these conditions were therefore not modeled using regression analyses.

#### Victim of deliberate harm

The average rating of shame when the victim of deliberate harm was significantly associated with the GPTS (*r* = 0.32, *p* = 0.002), PS (*r* = 0.32, *p* = 0.002), PDI (*r* = 0.42, *p* < 0.001), CAPS (*r* = 0.22, *p* = 0.04), CF (*r* = −0.22, *p* = 0.04), and BDI-II (*r* = 0.34, *p* = 0.001); the average rating of guilt when the victim of deliberate harm was significantly associated with the GPTS (*r* = 0.25, *p* = 0.02), PS (*r* = 0.28, *p* = 0.008), CAPS (*r* = 0.26, *p* = 0.02), CF (*r* = −0.26, *p* = 0.01), and BDI-II (*r* = 0.37, *p* < 0.001). These measures were entered as predictor variables in subsequent regression analyses.

Regression analyses are presented in Table 2. **Shame:** The first model (Model 1; paranoia/psychosis measures) accounted for 45% of the variance in shameful feelings and was significant, *F*_(4, 84)_ = 5.21, *p* = 0.001. Delusional ideation significantly predicted shame^1^, β = 0.35, *t* = 2.62, *p* = 0.01. The final model (Model 2; now including cognitive flexibility and depression) accounted for 46% of the variance in shameful feelings and was also significant, *F*_(6, 82)_ = 3.72, *p* = 0.003. Delusional ideation again predicted shame, β = 0.31, *t* = 2.35, *p* = 0.02. **Guilt:** Model 1 (paranoia/psychosis measures) accounted for 32% of the variance in guilty feelings and was significant, *F*_(3, 85)_ = 3.42, *p* = 0.02. However, no variable made an independent contribution. The final model (now including cognitive flexibility and depression) accounted for 41% of the variance and was also significant, *F*_(5, 83)_ = 3.26, *p* = 0.01. No variable made an independent contribution.

**Table 2:** Hierarchical multiple regression analyses entering shameful feelings when the victim of deliberate harm as the dependent variable (Moral Emotions task).

**Model**	**Predictor**	**β**	***t***	***p***	**95% CI**	**Partial correlation**	**R-Squared**
1 Shame (Victim of deliberate harm)	GPTS	0.17	1.33	0.19	−0.004, 0.02	0.14	
	PS	−0.02	−0.14	0.89	−0.03, 0.02	−0.02	
	**PDI**	**0.35**	**2.62**	**0.01**	**0.003, 0.02**	**0.28**	0.45
	CAPS	0.02	0.15	0.88	−0.05, 0.05	0.02	
2 Shame (Victim of deliberate harm)	GPTS	0.15	1.15	0.25	−0.01, 0.02	0.13	
	PS	−0.07	−0.46	0.65	−0.03, 0.02	−0.05	
	**PDI**	**0.32**	**2.35**	**0.02**	**.002, 0.02**	**0.25**	0.46
	CAPS	−0.02	−0.15	0.64	−0.06, 0.05	−0.02	
	CF	−0.05	−0.47	0.64	−0.04, 0.03	−0.05	
	BDI	0.14	1.07	0.29	−0.02, 0.07	0.12	

*GPTS, Green Paranoid Thoughts Scale; PS, Paranoia Scale; PDI, Peters' Delusions Inventory; CAPS, Cardiff Perceptions Inventory; CF, Cognitive Flexibility Scale; BDI-II, Beck Depression Inventory. Bold indicates a significant predictor*.

#### Victim of accidental harm

**Shame:** The average rating of shame when the victim of accidental harm was significantly associated with the GPTS (*r* = 0.24, *p* = 0.04) and CAPS (*r* = 0.22, *p* = 0.04). The model was significant, *F*_(2,86)_ = 3.75, *p* = 0.03 and accounted for 28% of the variance. However, neither variable made independent contributions. **Guilt:** The average rating of guilt when the victim of accidental harm was significantly associated with the GPTS (*r* = 0.29, *p* = 0.007), PS (*r* = 0.22, *p* = 0.04), and BDI (*r* = 0.28, *p* = 0.006). Regression analyses revealed that both models were significant [Model 1: *F*_(2, 86)_ = 3.89, *p* = 0.02 and Model 2: *F*_(3, 85)_ = 3.68, *p* = 0.02], accounting for 28% and 33% of the variance, respectively. Again, no variables made independent contributions.

### Prisoner's dilemma

**Figure 3:**
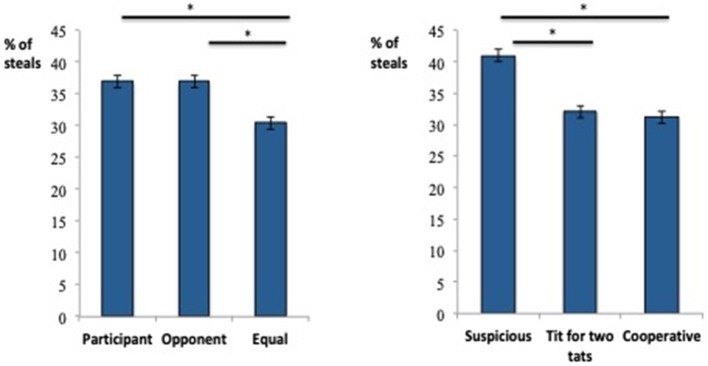
Main effects of Contribution type (left) and Player Strategy (right) on the Prisoner's Dilemma. *Indicates a significant difference between means.

The percentage of steals was calculated as the number of trials that the participant chose to steal from their opponent from the total number of trials across each strategy type. The Contribution (Participant contributed more, Opponent contributed more, Equal contributions) × Strategy (Suspicious, Tit for two tats, Cooperative) interaction was not significant, *F*_(4, 84)_ = 0.22, *p* = 0.93. However, there was a main effect of Contribution, *F*_(2, 86)_ = 8.15, *p* = 0.001, partial η^2^ = 0.16 (Participant contributed more: *M* = 36.95%, *SD* = 0.33; Opponent contributed more: 36.97%; *SD* = 0.33; Equal contributions: *M* = 30.43%, *SD* = 0.30; Figure 3), such that the percentage of steals was significantly less when the participant and opponent contributed equally [Equal contributions vs. Participant, *t*_(87)_ = 3.65, *p* < 0.001; Equal contributions vs. Opponent, *t*_(87)_ = 3.02, *p* = 0.003]. The percentage of steals between the participant and opponent contributions was not significant (*p* = 0.99).

There was also a main effect of Strategy, *F*_(4, 84)_ = 6.90, *p* = 0.002, partial η^2^ = 0.14 (Suspicious player: *M* = 40.96%, *SD* = 0.33; Tit for two tats player: *M* = 32.07%, *SD* = 0.37; Cooperative player: *M* = 31.19%, *SD* = 0.34; Figure 3), such that the highest percentage of steals was taken from the suspicious opponent [Cooperative vs. Suspicious, *t*_(88)_ = 2.58, *p* = 0.001; Tit for two tats vs. Suspicious, *t*_(87)_ = 3.73, *p* < 0.001]. The percentage of steals between the cooperative and tit for two tat strategies was not significant (*p* = 0.66; Figure 3).

Correlational analyses revealed that the percentage of steals made when the opponent cooperated was positively associated with paranoid thinking (GPTS; *r* = 0.22, *p* = 0.04).

### Ultimatum game

A repeated-measures ANOVA with the factors Contribution (Participant contributed more, Opponent contributed more, Equal contributions) and Offer (10, 20, 25, 30, 35, 40, and 50%) for acceptance rates revealed a significant interaction, *F*_(12, 77)_ = 105.76, *p* < 0.001, partial η^2^ = 0.94. There was a main effect of Contribution, F_(2, 87)_ = 50.78, p < 0.001, partial η^2^ = 0.54 (Opponent contributed more: *M* = 69.02%, *SD* = 0.23; Participant contributed more: *M* = 54.01%, *SD* = 0.31; Equal contributions: *M* = 52.21%, *SD* = 0.27), such that the percentage of acceptance was highest when the opponent contributed more [Opponent vs. Participant, *t*_(88)_ = 7.14, *p* < 0.001; Opponent vs. Equal contribution, *t*_(88)_ = 9.84, *p* < 0.001]. The percentages of acceptance between the participant's contribution and equal contributions were not significantly different (*p* = 0.11).

**Figure 4:**
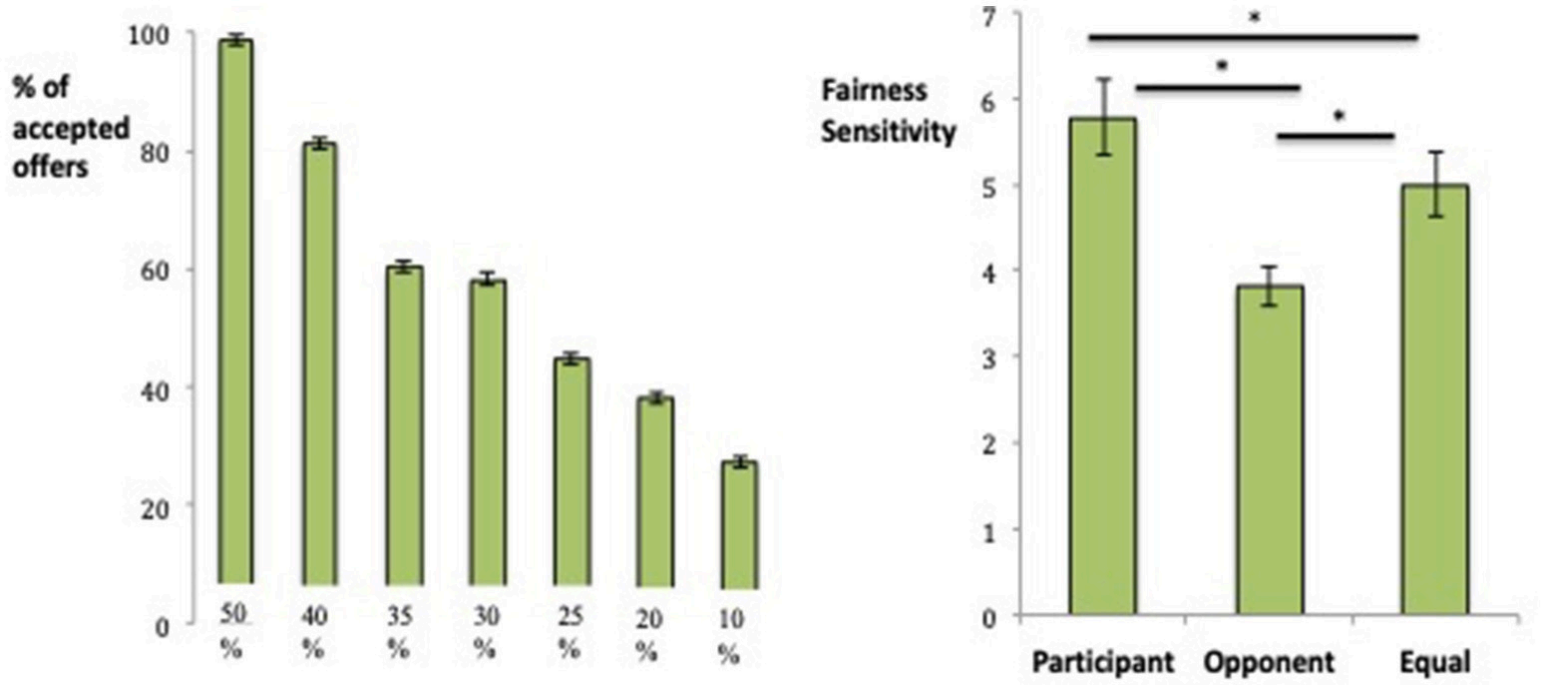
Main effect of Offer at each level of fairness (left) and fairness sensitivity for each Contribution type (right) on the Ultimatum Game. *Indicates a significant difference between means.

As expected, there was also a main effect of Offer, *F*_(6, 83)_ = 63.53, *p* < 0.001, partial η^2^ = 0.82, with the percentage of acceptance increasing monotonically as the offer increased (i.e., became more fair: 50%: *M* = 98.88%, *SD* = 0.06; 40%: *M* = 81.27%, *SD* = 0.26; 35%: *M* = 60.21%, *SD* = 0.27; 30%: *M* = 58.24%, *SD* = 0.35; 25%: *M* = 44.76%, *SD* = 0.39; 20%: *M* = 38.20%, *SD* = 0.39; 10%: *M* = 27.34%, *SD* = 0.43; Figure 4). All adjacent conditions (with the exception of 65% to 75%) significantly differed from each other (all *p*'s < 0.001).

To interpret the Contribution x Offer interaction, offer sensitivity was calculated as a measure of the degree to which participants increased their inclination to accept the offer as the amount proposed by the avatar increased. Offer sensitivity was calculated for each Contribution type using the following formula: *Offer sensitivity* = [2^*^acceptance at 50% offer] + [1^*^acceptance at 40% offer] + [0^*^acceptance at 30% offer]–[1^*^acceptance at 20% offer]–[2^*^acceptance at 10% offer]/Average offer. There was a main effect of Contribution, *F*_(2, 85)_ = 15.74, *p* < 0.001, partial η^2^ = 0.27. Sensitivity to fairness was greatest when the participant contributed more (Participant contributed more: *M* = 5.78, *SD* = 4.13; Opponent contributed more: *M* = 3.81, *SD* = 2.19; Equal contributions: *M* = 5.00, *SD* = 3.52; Figure 4), which significantly differed from when the opponent contributed more, *t*_(86)_ = 5.69, *p* < 0.001 and when equal contributions were made, *t*_(86)_ = 2.81, *p* = 0.006. Offer sensitivities between the opponent contribution and equal contributions were also significantly different, *t*_(88)_ = 4.27, *p* < 0.001.

Correlational analyses revealed that the overall average percentage of steals on the Prisoner's Dilemma (*M* = 34.72%, *SD* = 0.26) was negatively associated with the overall average percentage of offers accepted on the Ultimatum Game across conditions (*M* = 58.41%, *SD* = 0.26), *r* = −0.26, *p* = 0.02.

## Discussion

We used a single-trait approach to investigate the effects of paranoia on moral emotional processing and social decision-making, two social cognitive processes involving the perceptions of others intentions, in healthy participants with natural variation in paranoid thinking. We hypothesized that paranoid thinking would predict shameful feelings when the victim of deliberate (but not accidental) harm on the Moral Emotions task. We also hypothesized that distrust-based punishment behavior would be greatest when playing against a suspicious opponent on the Prisoner's Dilemma, and that stealing would be associated with paranoid thinking. Finally, we hypothesized that sensitivity to fairness would not be impaired on the Ultimatum Game.

### Emotional moral processing

Regression analyses offered some support for hypothesis one, revealing that almost half of the variance in shameful feelings was predicted by delusional ideation when the victim of intentional harm by another person. This effect was maintained even when including measures of depression and cognitive flexibility in the model. Although we expected paranoid thinking to be the key predictor, correlational analyses confirmed that trait paranoia and delusion vulnerability were moderately associated, as expected. In line with our hypothesis, no trait measures were significantly associated with either moral emotion when the victimiser of accidental or deliberate harm. However, trait measures relevant to paranoia and psychosis were positively associated with moral emotions when the victim of a harmful action, thus supporting that paranoia is specific to the experience of harm by others. This is consistent with previous studies showing that victimization (i.e., “he/she punished me, so I must have done something wrong”) is a key social risk factor for increased vulnerability to psychosis (20, 50). It is also likely that perceived social rank, particularly if viewing oneself in a lower out-group from the majority, exacerbates feelings of inferiority in those with pre-existing paranoia (32).

Cognitive models of persecutory delusions implicate altered emotional processes in their persistence (9, 10). For example, negative self-evaluations have been shown to be associated with positive symptoms in patients with schizophrenia (51) as well as with paranoia in the general population (52). Delusional beliefs may be particularly resistant to change if they are held in congruence with negative self-schemas (9). Shameful feelings in response to deliberate harm are more consistent with activation of negative schemas about the self (“Bad Me” paranoia) rather than the protection against distress from others (“Poor Me” paranoia) (53). Shame is strongly associated with the frequency and distress of paranoid thinking in patients with psychotic disorders (54), although less is known about its relationship with the content of delusions. As the PDI contains some items of persecution, suspiciousness, and paranoid ideas, it is possible that multidimensional delusional beliefs, including thoughts of persecution, predict shame in response to interpersonal harm. As no trait measure significantly predicted feelings of guilt, our data further suggest that, although conceptually related, negative moral emotions may have distinct manifestations based on different traits or vulnerability to specific pathology. Whereas guilt denotes a more depressive style of thinking, shame implicates other people and is more likely to precipitate self-referential processing when believing that one is the target of hostile actions. It is worth noting that we did not ask participants to make causal attributions of the amoral behavior depicted in the scenarios. Shameful feelings may activate negative self-evaluations that one deserves to be persecuted, thus leading to more internal attributions for negative outcomes (20, 55). However, it is also possible that attributions for harm by a perpetrator might directly contrast with emotional response, such that individuals with paranoia would externalize negative events to the victimiser or situation, but still respond in a self-devalued manner [i.e., one is both threatened and weak; (52)]. Although these possibilities cannot be addressed by the current study, findings from this task extend emotional processes implicated in cognitive models of delusions to moral emotions, in which shame may have particular relevance to perceived deservedness in those with elevated paranoia.

### Social decision-making

Both the Prisoner's Dilemma and Ultimatum Game allowed for the systematic manipulation of an opponent's behavior to enable investigation of cooperation and punishment during social economic exchange games (33). We found that uncooperative behavior on the Prisoner's Dilemma was significantly greater both when the participant and opponent contributed more in comparison with when the participant and opponent contributed equally, possibly reflecting protection of one's own contribution or a sense of entitlement for a larger share of the earnings. The percentage of steals between the participant's and opponent's contributions was not significantly different. This may reflect participants “predicting” that the opponent player would steal from them when the opponent contributed more, thus choosing to inflict punishment at the same tendency as when the participant contributed more, despite a loss in earnings for both players (i.e., participants would rather forfeit all gains then let their opponent succeed). In support of hypothesis two, we found more distrust-based punishment behavior when the opponent used a suspicious strategy of gameplay. In addition, more stealing was associated with higher levels of paranoid thinking, but, somewhat unexpectedly, only when playing against a *cooperative* opponent. Others have shown a positive association between paranoia and choice to compete on this task in the general population, suggesting a behavioral marker of non-clinical paranoia (31). Here, distrust-based punishment behavior was greatest when playing against a suspicious opponent, but paranoid thinking was associated with stealing from the player who always chose to split, thus showing expectation of (and an inability to update beliefs about) unfounded malevolent intentions of another person, despite their full cooperation toward mutually advantageous reward.

Results from the Ultimatum Game supported hypothesis three. It was found that, consistent with previous studies in the healthy population (33, 56, 57), participants generally rejected unfair offers, but accepted significantly more unequal offers when the opponent contributed more. Furthermore, sensitivity to fairness was greatest when the participant contributed the most. There has been some evidence that patients with schizophrenia are less averse to unfairness to their own disadvantage (36), although others have suggested that impaired decision-making may be specific to the presence of psychopathology, symptom severity (either negative and/or cognitive impairments in working memory and executive function), or disrupted connectivity in emotion-related areas of the brain including the anterior cingulate cortex, orbitofrontal cortex, and amygdala (38, 39, 58). Future studies comparing healthy individuals with subclinical paranoia and patients with schizophrenia would help elucidate at what level of severity these possibilities compromise sensitivity to fairness. Lastly, it was found that participants who were more likely to steal on the Prisoner's Dilemma were less likely to accept offers on the Ultimatum Game, thus demonstrating an inverse relationship between cooperation and assertiveness. We note that the relationship between cooperative behavior and reasoning biases are relatively under-investigated in paranoia and suggest that introducing monetary incentives distinguishes reasoning (using the information available to draw inferences) from decision-making (selecting the best option at different levels of risk), as shown in the socioeconomic strategies probed here.

### Implications and conclusions

Overall, the key findings from this study are, firstly, that delusional ideation predicts shameful feelings when the victim of deliberate harm by another person; secondly, that distrust-based punishment behavior is greatest in response to a suspicious opponent, but that inflicting punishment on a cooperative opponent relates to increased paranoid thinking; and thirdly, that sensitivity to fairness remains intact when economically disadvantaged. As this study included relatively young adults, future studies should replicate these findings in samples better representative of patients with schizophrenia. Use of virtual reality methods and an actual or confederate opponent (rather than avatar) would also improve the genuineness of social interactions involving the perception of the intentions of others. Clinical implications include increased specificity of impaired emotional processes in cognitive models of threat beliefs (9, 10), in which shameful feelings in response to deliberate harm were shown to be one type of negative self-evaluation predicted by delusion proneness. Furthermore, expectation of treatment, which differs on the basis of one's perceived social rank (e.g., feelings of inferiority irrespective of other people's intentions, hostile, or not), is likely to precipitate social interactions with negative outcomes. For example, punishing a suspicious opponent may be advantageous, but paranoid thoughts associated with punishing a cooperative opponent will have implications for reduced pro-social behavior. Expectation of unfair treatment is also likely to decrease self-reflection about one's own worth, which in turn decreases ability to understand the intentions of others (59). Interventions that target social-cognitive deficits [Social Cognition and Interaction Training; (60)], pre-existing biased cognitive mechanisms [e.g., Cognitive Bias Modification for paranoia, CBM-pa; (61)] and metacognition [Metacognitive training, MCT; (62)] including difficulties making sense of the mental states of others [Metacognitive Interpersonal Therapy; (63)] are key approaches for improving social and cognitive outcomes in patients with schizophrenia that act directly on affective domains or modify related underlying cognitive-affective biases. Such interventions may also have a useful application for reducing the social-cognitive effects of paranoia and delusional capacity in the general population. Finally, this study further validated EMOTICOM as a useful neuropsychological battery for assessing affective cognition in non-psychiatric samples (22). The effects of paranoia on social cognitive processes including moral emotions and decision-making warrant further investigation using EMOTICOM at more severe levels of persecutory thinking.

## Author contributions

GS conceived and designed the study, obtained ethical approval, collected data, analyzed and interpreted data and wrote the manuscript. HJ, NR, SK, and AZ collected data. TR and BS interpreted data and provided feedback on the manuscript.

### Conflict of interest statement

TR consults for Cambridge Cognition, Lundbeck, Mundipharma, and Unilever and receives royalties for CANTAB from Cambridge Cognition and editorial honoraria from Springer-Verlag and Elsevier. BS consults for Cambridge Cognition, Peak and Mundipharma. The remaining authors declare that the research was conducted in the absence of any commercial or financial relationships that could be construed as a potential conflict of interest.

## Footnotes

^1^Correlational analyses revealed that mean scores on the PDI and GPTS were significantly associated, *r* = 0.45, *p* < 0.001. Scores on the PDI ranged from 0 – 135.
